# Immunomodulatory Role of Capsular Polysaccharides Constituents of *Cryptococcus neoformans*

**DOI:** 10.3389/fmed.2019.00129

**Published:** 2019-06-19

**Authors:** Debora Decote-Ricardo, Isabel Ferreira LaRocque-de-Freitas, Juliana Dutra B. Rocha, Danielle O. Nascimento, Marise P. Nunes, Alexandre Morrot, Leonardo Freire-de-Lima, Jose Osvaldo Previato, Lucia Mendonça-Previato, Celio Geraldo Freire-de-Lima

**Affiliations:** ^1^Instituto de Veterinária, Universidade Federal Rural do Rio de Janeiro, Seropédica, Brazil; ^2^Instituto de Biofísica Carlos Chagas Filho, Universidade Federal do Rio de Janeiro, Rio de Janeiro, Brazil; ^3^Laboratório de Imunoparasitologia, Instituto Oswaldo Cruz, FIOCRUZ, Rio de Janeiro, Brazil; ^4^Faculdade de Medicina, Universidade Federal do Rio de Janeiro, Rio de Janeiro, Brazil

**Keywords:** *Cryptococcus neoformans*, glucuronoxylomannan (GXM), galactoxylomannan (GalXM), immunomodulation, immuno evasion

## Abstract

Cryptococcosis is a systemic fungal infection caused by *Cryptococcus neoformans*. In immunocompetent patients, cryptococcal infection is often confined to the lungs. In immunocompromised individuals, *C. neoformans* may cause life-threatening illness, either from novel exposure or through reactivation of a previously acquired latent infection. For example, cryptococcal meningitis is a severe clinical disease that can manifest in people that are immunocompromised due to AIDS. The major constituents of the *Cryptococcus* polysaccharide capsule, glucuronoxylomannan (GXM), and galactoxylomannan (GalXM), also known as glucuronoxylomanogalactan (GXMGal), are considered the primary virulence factors of *Cryptococcus*. Despite the predominance of GXM in the polysaccharide capsule, GalXM has more robust immunomodulatory effects on host cellular immunity. This review summarizes current knowledge regarding host-*Crytococcus neoformans* interactions and the role of capsular polysaccharides in host immunomodulation. Future studies will likely facilitate a better understanding of the mechanisms involved in antigenic recognition and host immune response to *C. neoformans* and lead to the development of new therapeutic pathways for cryptococcal infection.

## Introduction

In *Cryptococcus neoformans*, capsular polysaccharides are located externally to the cell wall. The polysaccharides constituting the capsule can be found attached to the cell wall or released into the environment, in which case they are termed exopolysaccharides. Biochemical studies have shown that the *C. neoformans* capsule primarily comprises glucuronoxylomannan (GXM), representing ~88% of the capsule. GXM is a polymer that consists mostly of a linear α-(1–3)-mannan substituted with β-(1–2)-glucopyranosyluronic acid and β-(1–4)-xylopyranosyl. The *C. neoformans* capsule also comprises 10% galactoxylomannan (GalXM), also called glucuronoxylomannogalactan (GXMGal) and 2% mannoproteins. The GalXM consists of an α-(1 → 6)-galactan backbone with galactomannan side chains that are further substituted with variable numbers of xylose and glucuronic acid residues (1). It has recently been characterized that the polysaccharide capsule can function as a flotation device to facilitate transport and dispersion in aqueous fluids (2).

It is known that, the manifestation and severity of cryptococcal infections, as well as the clinical presentation of the disease depend on the immune response of the patient (3, 4). Clinical and experimental studies have suggested that cryptococcosis is controlled by cell-mediated immunity in immunocompetent patients who develop a Th1 response (5, 6). In addition, the production of inflammatory mediators induces the death of *C. neoformans* potentiating the antimicrobial activity of immune cells (7–12).

The Involvement of the cellular-mediated immune response in the cryptococcosis has been described. Macrophages and dendritic cells are related in the recognition, phagocytosis, and presentation of antigens and activation of the host response (13, 14). The involvement of the NK cells has been characterized in response to the *C. neoformans*; after the recognition via β-integrins, cytolytic degranulation occurs leading to the death of fungal cells in a manner similar to that observed in tumor cells (15, 16). Despite the importance of the immune response in the *C. neoformans* infection, it has recently been described that the sudden increase of this response could cause non-specific symptoms favoring tissue damage; this phenomenon is known as inflammatory response syndrome (IRIS) (17–21). In some severe cases, medications such as corticosteroids are required to suppress inflammatory response until resolution of infection (22, 23).

## Innate Response to *C. neoformans*

Initial infection of *C. neoformans* occurs via inhalation of infectious propagules and subsequent colonization of the respiratory tract. Macrophages and dendritic cells phagocytize the opsonized fungi. *C. neoformans* and their secreted products can be recognized by many cellular receptors, including the mannose receptors CD14, TLR2, TLR4, and CD18 (13, 24–27). The TLR-associated adapter protein, MyD88, is known to play an important role in immune response to *C. neoformans*, as mice deficient in MyD88 have increased susceptibility to Cryptococcosis (27–29). Macrophages are important for defense against *Cryptococcus* and respond to *C. neoformans* infection by releasing pro-inflammatory signals such as the chemokines IL-1α, and IL-1β. Furthermore, mice that do not encode the IL1R1 receptor, the expression of which is stimulated through MyD88, exhibit more disease phenotypes when compared to the wild type mice (30, 31).

In addition to phagocytosis, the killing of *C. neoformans* is crucial for successful host response. The internalization of viable forms may facilitate the dissemination of this microorganism to different parts of the vertebrate host. Macrophages become efficient effector cells when stimulated by pro-inflammatory cytokines, increasing microbicidal mechanisms, including lysosomal fusion, acidophagy of phagosomes, iron sequestration, and enzymatic degradation of proteins. Extracellular killing of the yeast is mediated by the release of cytokines, NO, and ROS (32–34). *C. neoformans* can also be sequestered in tissues by multinucleated giant cells (35). Moreover, it has recently been demonstrated that extracellular vesicles of *C. neoformans* can stimulate macrophages, which increases its antimicrobial activity and induces direct death of the fungus (36).

Similar to bacteria, fungi have developed strategies to survive within phagocytes. Most of these pathogens kill the host cell to escape and infect other tissues (37, 38). It has recently been suggested that *C. neoformans* can exploit monocytes and macrophages by using these cells as “Trojan horses,” allowing them to disseminate throughout the host without being detected by the immune system (38–40). Tucker and Casadevall have shown that opsonized *C. neoformans* cells are capable of being internalized in <2 min by alveolar macrophages or J774 lineage (41). However, infected macrophages are unable to maintain the acidity of the phagolysosome, which results in phagolysosome membrane lysis, *C. neoformans* replication, concomitant release of the polysaccharides, and eventual lysis of infected macrophages. Complementing Tucker and Casadevall's work, a later study observed lysis of alveolar epithelial cells 18 h after infection with *C. neoformans* (42). Subsequently, it was demonstrated that *C. neoformans* can also escape from macrophages through a mechanism that does not result in cell death (43). After the onset of intracellular replication, *C. neoformans* are able to extrude from the macrophage phagosome into extracellular space, an event that has been termed “phagosome extrusion,” Phagosome extrusion involves the formation of large fungal cell-filled phagosomes followed by phagosome ejection and subsequent survival and further replication of the fungal cells in the macrophage extracellular space (44). This data suggests that non-lytic expulsion of *C. neoformans* is a phenomenon that may have significant clinical implications in understanding the invasion of the central nervous system by cells that serve as “Trojan horses” for *C. neoformans* (40, 45–47).

The development of cell-mediated protective immunity against *C. neoformans* depends, in part, on the activation of dendritic cells by the fungus (48). Dendritic cells are also important for antifungal immunity, as they can recognize and phagocyte different fungi, such as *C. neoformans, Aspergillus fumigatus, Candida albicans*, and *Histoplasma capsulatum* (49–53). Mannose and FcγRII receptors present on dendritic cells are essential for *C. neoformans* phagocytose and antigen presentation to T cells (54). After dendritic cells identify and phagocytize *C. neoformans*, they express costimulatory molecules, migrate to the lymphoid tissues, and secrete cytokines (53, 55, 56). After real-time imaging, Wozniak and Levitz demonstrated that *C. neoformans* can be found in lysosomal compartments 20 min after the onset of phagocytosis, resulting in the degradation of the fungal cells followed by antigen presentation to T cells (57).

The development of cell-mediated protective immunity against *C. neoformans* depends, in part, on the activation of dendritic cells by the fungus (48). Dendritic cells also represent an important interface between innate and adaptive immunity (58–60). In addition, the interaction between dendritic cells and *C. neoformans* modulates T cell responses *in vitro* (61–63). *In vivo* observations have determined that T cells migrate to the thoracic lymph nodes after infection (64–66). In addition, both dendritic cells and CD4+ T cells were detected in bronchoalveolar infiltrate from lungs of *C. neoformans* infected mice. This phenomenon coincided with increased expression of IL-12 cytokines from both subunits (p40 and p35) and the presence of IFN-γ in the lung homogenate (67).

## Adaptive Response to *C. neoformans*

*C. neoformans* infection is normally controlled by cell-mediated immunity in immunocompetent patients who develop a Th1 response following the production of IL-12, TNF-α, IFN-γ, and NO (5, 10, 68–70). CD4+T cells producing IFN-γ (71) are important for inducing immune defenses that control the growth and propagation of *C. neoformans* (70, 72). The formation of granulomas also serves as an important antifungal mechanism (73, 74). In addition to CD4+ T cells, granulomas also contain macrophages, NK cells, and CD8+ T cells (73). Effector NK cells and CD8+ T cells are potent producers of perforin and granulysin that destroy cells infected with *C. neoformans* and directly destroy the fungus itself (75, 76). Recent studies with the cryptococcosis murine model have demonstrated that Th1 and Th17 subtype responses are associated with disease protection and Th2 responses are deleterious for the host (77–79).

## Immunomodulatory Role of Capsular Constituents

Capsules are commonly found in bacteria. However, the only pathogenic encapsulated fungus described so far is *Cryptococcus*. In this model, the capsule is the most studied structure since it is considered one of the main virulence factors. When in the saprophytic phase, the polysaccharide capsule protects the fungus against various types of stress and dehydration. In the murine experimental model, acapsular mutants do not cause cryptococcosis or have reduced virulence. Other studies have demonstrated that the capsular polysaccharides GXM and GalXM, have immunomodulatory properties and improve fungal survival by aiding host immune evasion (80). In addition, the fungus releases constituents of the polysaccharide capsule which can easily be found in body fluids during infection (81). These capsular constituents exert various immunomodulatory roles in host cells and are been used as research models by several research groups.

## Immunomodulatory Activity of GXM

*C. neoformans* is considered an opportunistic intracellular pathogen (82) and, as mentioned previously, can survive, and even replicate inside macrophages. The role of the capsule in intracellular parasitism was demonstrated mostly using the CAP67 mutant, which does not contain GXM in the capsule and is not able to replicate within phagocytic cells (83). This indicates that the capsule, particularly GXM, plays a key role in intracellular parasitism. GXM is also involved in the inhibition of phagocytosis probably due to the fact that this molecule is polyanionic, which may cause an electrostatic repulsion that prevents host immune cells from interacting with and eliminating fungal cells (84). Inhibition of phagocytic events will also decrease the amount of presented antigen to T cell and, consequently, reduces host immune response (85). A GXM molecule could also present distinct combinations of motifs, demonstrating the complex heteropolymeric structure of this capsular polysaccharide. Recently, the presence of a secreted lactonohydrolase (LHC1) of *C. neoformans*, has been characterized in the formation of a higher order capsular structure proposing enzymatic remodeling after assembly of polysaccharides in the extracellular space and thus inhibiting the deposition of components of the complement system, thus avoiding the destruction of the fungus by formation of the membrane attack complex and the formation of fragments iC3b, which are important opsonic (86).

Several research groups have suggested that GXM behaves as a molecule that contributes to immune suppression. Syme et al. (54) demonstrated that GXM is directly involved with FcRγII expression in monocytes, macrophages, and dendritic cells, which results in an inhibitory signal that suppresses host immune response. GXM also induces secretion of IL-10 and IL-8 in human monocytes and neutrophils (87, 88). IL-10 is a potent suppressor of proinflammatory cytokines, which suggests that *C. neoformans*-induced IL-10 secretion interferes with cell-mediated immunity (89). In 2001, GXM was reported to indirectly reduce IL-12 secretion in monocytes by directly increasing IL-10 secretion in monocytes co-cultured with T cells (71). GXM also induces the accumulation of TGF-β in macrophages (90). In contrast to mice infected with a hypocapsular mutant, mice infected with encapsulated strains were not able to produce Th1 cytokines, due to the accumulation of significant amounts of IL-10. These results suggest that yeasts encapsulated with GXM are able to inhibit the development of a Th1-type protective response via the induction of IL-10 (71, 91). Despite this, the immunosuppressive activity of GXM has beneficial effects in other types of inflammatory pathologies. GXM can act as a potent immunosuppressant during induced arthritis (92) and inhibits NETs production in neutrophils (93).

In addition to having potent immunosuppressive effects on cytokine production, GXM is also capable of inducing apoptosis in different systems. Monari et al. (94) have demonstrated that Fas-L expression in macrophages induces apoptosis of activated T lymphocytes. In 2008, the same group demonstrated that apoptosis of T lymphocytes involves intrinsic and extrinsic pathways (95). Furthermore, our group observed that capsular GXM is able to induce apoptosis in macrophages through a mechanism that involves the increased production of Fas and Fas-L (90). This ability of GXM expands the repertoire of immunosuppressive activities observed during cryptococcosis pathogenesis and complements other reports in literature, where Fas-induced apoptosis has been described in infections caused by other microorganisms (73, 96). The mechanism of GXM-induced cell death may also be related to inhibition of glycolytic flux (97).

Chiapello et al. (98) demonstrated that GXM is capable of inducing cellular apoptosis in different organs after treatment. Apoptosis was also observed *in vitro* in rat splenocytes (99). The authors suggested that apoptosis would be a consequence of an immunomodulation of the host defense during infection. GXM deposition in tissue, increased splenocyte production and release of the anti-inflammatory cytokines may lead to these processes (98–100). In rats infected with *C. neoformans*, T cell production of IL-10 could reduce LFA-1 expression and IL-2 production, resulting in decreased T-cell activation and proliferation capacity. Chiapello et al. (98) also demonstrated that total splenocytes from infected mice and activated by concanavalin A (ConA), increased IL-10 production. Addition of anti-IL-10 antibody reversed the suppressor effect, suggesting that this cytokine would be related to suppress total splenocyte proliferation (100). Thus, the production of IL-10 during cryptococcosis could prevent the activation of T cells-mediated control of *C. neoformans*, resulting in the spread of the disease. These observations confirm other studies that have demonstrated that pathogen-derived molecules promote apoptosis in the host cell, and thus they may be important factors in the survival of infectious microorganisms (101, 102).

## Immunomodulatory Activity of GalXM

The majority of studies on the immunomodulatory effects of capsular polysaccharide from *C. neoformans* has been performed with GXM. There are fewer studies demonstrating the immunomodulatory functions of GalXM, but they have increased in recent years. Chaka et al. (103) demonstrated that GalXM is capable of inducing the production of TNF-α in peripheral blood mononuclear cells (PBMC). In addition, our group has observed that GalXM induces TNF-α release and iNOS expression, which results in NO production (90).

Delfino et al. (104) showed that GalXM induces IL-6 release by monocytes. In 2006, another group demonstrated that prolonged stimulation with the CAP67 mutant increases expression of IL-6 in PBMC and results in resolution of infection, whereas that does not occur encapsulated fungus (105). This suggests that GXM and GalXM have different immunomodulatory activities. Despite this, GalXM is also capable of inducing apoptosis in different cells of the immune response. Pericolini et al. (106) demonstrated that GalXM induces apoptosis in human T cells, at a rate ~50 times greater than GXM, via activation of caspase 8. This suggests that GalXM suppresses the proliferation of T cells. Our group also demonstrated that GalXM induces apoptosis in macrophages mediated by Fas/FasL interaction. This effect was greater than that observed by GXM (90). GalXM-mediated cell death could enhance the suppressive effect of GXM during cryptococcosis.

Galactose, a major component of GalXM, has been described as an important virulence factor of *C. neoformans* (107). The enzymes UDP-Glc epimerase and UDP-Gal transporter participate in GalXM biosynthesis and are present in GXM positive and GalXM negative phenotypes. Moyrand et al. demonstrated that strains encoding mutated forms of the enzymes UDP-Glc epimerase (uge1Δ) and UDP-Gal transporter (ugt1Δ) are unable to colonize the brain in the first days of infection. These results suggest that although these constituent polysaccharides have different profiles, they may act together to suppress the host's immune response during infection. Recently, it was demonstrated that treatment with GalXM activates dendritic cells and induces Th17 differentiation when co-cultured with T cells. The authors also demonstrated that treating mice with GalXM prior to infection induced immune protection and this phenomenon was dependent on IL-6 and IL-17 production (108).

## The Importance of Xylosylation

UDP-Xyl is the substrate for the synthesis of xylose-containing glycoconjugates (109). In *C. neoformans* xylose is present in the capsular constituents GXM and GalXM and in yeast membrane glycoinositolphosphorylceramides (GIPCs) (110, 111). The enzyme β-1,2-xylosyltransferase transfers xylose to α-1,3-dimannoside to form the sequence Xyl-β-1,2-α-1,3-Man. In a *cxt1* mutant strain of *C. neoformans*, which is deficient in beta-xylosyltransferase, shows decrease or lack of xylose units in GalXM and GXM, respectively. This mutante strain presents low virulence in the murine model (111). Mutant strains that encode a deletion in the UDP-xyl synthase gene (uxs1) produce GXM without β(1,2)-xylose (112). The genes CAS31, CAS32, CAS33, CAS34, and CAS35 are homologous to CAS3 and help regulate the addition of xylose units onto the GXM side chain (113). Experiments using the Δuxs1 strain, which does not encode UDP-Xyl synthase, suggest that the presence of xylose units in the GXM structure contributes to *C. neoformans* virulence by delaying the deposition of the C3 complement system factor and altering the accumulation of GXM in the spleen (114). Moyrand et al. (112) observed that a cohort of mice infected with the Δuxs1 strain survived after 60 days of infection, whereas mice infected with the wild type strain died 30 days post-infection. Animals infected with Δuxs1 strain were sacrificed 90 days after infection and no *C. neoformans* colonies were observed in mouse brains. These results suggest the importance of xylosylation for *C. neoformans* virulence and infectivity. In addition, deletion of the *Cxt1* gene, which is responsible for encoding a β(1,2)-xylosyltransferase involved in capsule synthesis, led to lower levels of infection by *C. neoformans* in murine models of disease (115). These data suggests that xylose is important for basal interactions between the pathogen and the host (116).

## Conclusions

*C. neoformans* is an opportunistic fungus that has a polysaccharide capsule that exhibits important immunomodulatory activity *in vivo*. Both GXM and GalXM polysaccharides exert modulatory effects on different components of the innate and adaptive immune response. The function of all cell types is impacted by capsular polysaccharides, including macrophages which are important phagocytes and antigen-presenting cells that reside in various tissues throughout the body. The *C. neoformans* polysaccharides are important pathogen associated molecular patterns that are essential for stimulating cell-mediated immunity. However, there is no cell receptor that specifically recognizes these molecules. The cell-host recognition may occur through different receptors present on the cellular surface.

Further understanding the interactions between capsular polysaccharides and immune cells as well as the immunomodulatory effects of capsular polysaccharides may lead to the development of vaccines against this important pathogen.

## Author Contributions

DD-R, IL-d-F, JR, DN, MN, AM, LF-d-L, JP, LM-P, and CF-d-L: wrote the paper. All authors read and approved the final version of the manuscript.

### Conflict of Interest Statement

The authors declare that the research was conducted in the absence of any commercial or financial relationships that could be construed as a potential conflict of interest.
